# Added value of a specialized mobile palliative care team compared to the usual care in the primary care setting of Croatia: a 3-month prospective cohort study

**DOI:** 10.1186/s12904-026-02239-1

**Published:** 2026-07-25

**Authors:** Mirjana Huić, Darija Erčević, Eszter Saghy, Renata Grenković, Romana Tandara Haček, Maja Vajagić, Sanja Predavec, Marcell Csanádi, János G. Pitter, Marta Čivljak, Tina Poklepović Peričić, Livia Puljak, Ana Utrobičić, Maureen Rutten-van Mölken, Antal T. Zemplényi

**Affiliations:** 1 Department for Development, Research and Health Technology Assessment, Agency for Quality and Accreditation in Health Care and Social Welfare, Zagreb, Croatia; 2 Division of Pharmacoeconomics, Faculty of Pharmacy, University of Pécs, Pécs, Hungary; 3 Croatian Health Insurance Fund, Zagreb, Croatia; 4 Ministry of Health, Zagreb, Croatia; 5 Syreon Research Institute, Budapest, Hungary; 6 Faculty of Health Studies, Center for Evidence-Based Medicine, Catholic University of Croatia, Zagreb, Croatia; 7 University of Split School of Medicine, Split, Croatia; 8 University of Split, Split, Croatia; 9 Erasmus School of Health Policy and Management, Erasmus University Rotterdam, Rotterdam, the Netherlands; 10 Faculty of Health Studies, School of Medicine, Center for Evidence-Based Medicine, Catholic University of Croatia, Ilica 244, Zagreb, 10000 Croatia

**Keywords:** Multimorbidity, Integrated care models, Palliative care, Mobile multidisciplinary specialist palliative care team, Outpatient setting, Health/well-being, Experiences with care, Health-care costs

## Abstract

**Background:**

Croatia has been implementing a Mobile multidisciplinary specialist palliative care team (MMSPCT), a home-based specialized palliative care service at the primary healthcare level since 2011. This study aimed to evaluate whether a MMSPCT performs better than usual care, in terms of health/well-being, experience with care, and cost outcomes.

**Methods:**

A 3-month prospective cohort study was performed during 2017–2019. Two cohorts were compared: MMSPCT vs. usual care cohort. Sample size was based on the literature data, ranging 150–200 patients per group. A SELFIE questionnaire with selected standardised instruments was used to assess health/well-being, experience with care, and cost (on four categories: drugs, medical devices, hospitalisation for acute and chronic care), at the beginning (T0), after 1 (T1), and after 3 months (T2). Propensity score weighting using subclassification method was used to reduce selection bias by grouping exposed and control group patients into four subclasses based on similar propensity scores. The average treatment effect on the treated (ATT) was estimated using linear models with propensity score weighing and clustering based on the subclasses of propensity scores. Cost outcome variables were analysed using hurdle models due to zero-inflation in the data.

**Results:**

410 palliative care patients were included, 220 in MMSPCT and 190 in usual care cohort; 94 patients (42.7%) vs. 124 (65.3%) completed the study at 3-months follow-up. Baseline characteristics: age, gender and number of health conditions were balanced after propensity score weighting, but not living situation, education level, marital status and smoking. After 1 and 3 months, MMSPCT patients scored significantly better on person centeredness: 0.69 (3.83%) and 1.87 (10.39%) units higher score and compassionate care: 3.33 (2.78%) and 6.14 (5.12%) points more. After 3 months, they scored significantly better on physical (4.1 points more) and emotional functioning (5.18 points more) and had significantly lower measured healthcare costs. More MMSPCT patients died at their preferred place of death (48% vs. 23%).

**Conclusion:**

MMSPCT at primary care level may offer added value to palliative patients in Croatia and may result in a net saving in measured healthcare costs. Further clinical research is needed with a longer follow-up period and children’s cohorts.

**Supplementary Information:**

The online version contains supplementary material available at https://doi.org/10.1186/s12904-026-02239-1.

## Background

There are different models for the provision of home-based palliative care services. Literature data show mixed results of home-based end-of-life care on patient-reported health and well-being measures (PROMS), experience with care measures (PREMS), and costs [1]; taken together, they cover the Triple Aim [2].

Using the triple aim, it has been suggested that palliative care can be an effective means to achieve those goals and address the needs of patients [3]. To provide optimal palliative care, new palliative care interventions need to be continuously assessed and compared to the existing practice.

A Cochrane systematic review on home-based end-of-life care found that such care increased the likelihood of dying at home compared with usual care; it may slightly improve patient satisfaction at one-month follow-up, but the effect on patient health outcomes (functional status, psychological well-being, or cognitive status), including control of symptoms, the effect on caregivers, staff and health service costs was uncertain. Two highly important PREMS, person-centredness and compassionate care outcomes [4, 5], were not measured in trials included in that Cochrane review [6]. Person-centredness is ensuring that care matches an individual’s needs, capabilities and preferences and jointly making informed decisions [7]. It pertains to tailored care whereby care providers have a holistic understanding of the person’s problems, preferences, and needs. This care is respectful and supportive. There is good communication between patient- and provider, which forms the basis of shared decision-making. Compassionate care is defined as care provided in a warm, sensitive and dignified way with sympathy and respect [8].

When it comes to palliative and end-of-life care, there are noticeable differences between patient and proxy perspectives, which further underscore the need for personalized, patient-centered approaches which include early patient engagement in care planning [9]. Accordingly, healthcare personnel needs to advocate for patients, to support them in what they want, and to ask them what the best thing for them is [10]. It is crucial to develop sustainable and transferable models of palliative care delivery for different types of patients [11], and to test them in a research setting.

In Croatia, the Mobile multidisciplinary specialist palliative care team (MMSPCT) has been introduced as a home-based palliative care service at the primary healthcare level, based on the National Health Care Strategies (2012–2020 and 2021–2027) related to the integrated palliative care model [12, 13]. The first MMSPCT was established in Croatian Istria County in 2011, as a pilot county project, with a specialist in radiotherapy-oncology, two nurses as full-time permanent employees, and external part-time experts (like an oncologist, neurologist, surgeon, psychiatrist, physiatrist, dentist, psychologist, pharmacist and social worker). Between 2012 and 2015, they provided different interventions/procedures for palliative care patients at their homes, such as urethral and suprapubic catheter insertion, necrectomy, abdominal and pleural puncture, and parenteral therapy [14].

The first MMSPCT contracted by the Croatian Health Insurance Fund was established in 2016 in Varaždin County. The number of such teams in Croatia increased to thirty-three in 2020, as did the annual financial allocation for a standard mobile palliative team (from 440,314 HRK to 486,376 HRK) [15, 16]. The annual financial allocation for a standard family physician team was almost identical and increased from 475,570 HRK in 2017 to 484,552 HRK in 2020 [15–17].

Until our study, controlled studies in Croatia (such as randomised controlled trials or observational studies), were not conducted to evaluate effectiveness of MMSPCT in comparisons to the usual care at primary healthcare level, and to provide sufficient evidence to health policy decision makers on further MMSPCT implementation within the country.

As part of the EU-funded Sustainable intEgrated care models for multi-morbidity, delivery, FInancing and performance (SELFIE) project [18–20], our study aimed to evaluate whether an MMSPCT performs better in terms of health/well-being, experiences with care, and costs (corresponding to the ”triple aim”) compared to the usual care for palliative patients at primary healthcare level.

## Methods

### Study design

A 3-month prospective multi-site cohort study was performed including adult palliative patients who are referred to and treated by a MMSPCT (exposed cohort) or received usual care (control cohort) in primary care setting in Croatia. The study started in October 2017 and finished in May 2019. This study was conducted as one of the case studies in Horizon2020-project SELFIE [21].

### Ethics

Medical Ethics Review Committee of the Erasmus University Medical Center approved the study protocol for the SELFIE project (document no. MEC-2017-402; June 12, 2017). Ministry of Health of the Republic of Croatia endorsed the study protocol (December 29, 2016), as well as Ministry of Demography, Family, Youth and Social Policy of the Republic of Croatia (March 1, 2017). Subsequently, the study was approved by the Ethics Committee of each participating health care centre. All study participants provided informed written consent. The study was conducted in line with the Helsinki declaration and all applicable laws and regulations.

### Population

The study population consisted of a sample of palliative patients with multi-morbidity (around 60%-80% cancer patients and 20%-40% non-cancer patients) from six Croatian counties (three exposed to the MMSPCT: City of Zagreb, Istria County, Primorje-Gorski Kotar County, and three provided usual care: Karlovac County, Koprivnica-Križevci County and Zagreb County. In July 2018, Zagreb County started with MMSPCT teams and after this period contributed to the exposed group too.). A consecutive sampling method was used to recruit palliative patients for this research. Further inclusion and exclusion criteria were applied to palliative patients;

#### Inclusion criteria

Patients were considered for enrolment by the MMSPCT in the exposed group or by their primary care physicians in the control group, if they are palliative patients (according to the Supportive and Palliative Care Indicators Tool-SPICT™ and ICD-10: Z51.5), 18 years or older, with a life expectancy ranging from 1 to 6 months.

#### Exclusion criteria

Patients and/or families who refused care by the MMSPCT, who were not able to give answers in questionnaires (had a cognitive condition or were unresponsive or nonverbal), patients unlikely to survive more than 1-month based on their clinicians’ judgments and those who did not want to sign informed consent were excluded.

Treatment by the MMSPCT in the exposed group or usual care by the primary care physicians in the control group was discontinued when the patient died, was permanently transferred to the secondary health or social care institutions or no longer wanted this treatment. A patient could withdraw participation from this study at any time. If this happened in the exposed group, the primary care physician was informed as well.

#### Exposed cohort

Palliative patients treated by a MMSPCT, in three Croatian counties that already implemented it (The City of Zagreb County, Istria County and the Primorje-Gorski Kotar County, which cater to about 3000, 800 and 1000 palliative care patients each year, respectively).

An MMSPCT at the primary care level, as a minimum standard, consists of a physician-clinical specialist with education in palliative care and one nurse with education in palliative care; the rest of team could include additionally other physicians-clinical specialists in the team (i.e., oncologist, neurologist, psychiatrist, surgeon, dentist), psychologists, pharmacists, social worker, occupation therapist, physiotherapist, volunteer and priest. Services from other team members are provided upon request from the physician or nurse, representing the multidisciplinary and interdisciplinary work with other services in providing continuous and complete palliative care. MMSPCT starts to provide specialised palliative care service after request received by a primary care physician of family medicine, based on the 1st call of palliative patients or their families. After this first contact, MMSPCT provides specialised palliative care service based on direct calls received from palliative patients or their families and different palliative care needs.

During this study, MMSPCT did not yet provide full 24/7 care for palliative patients at their home and thus MMSPCTs were not available during the night. Apart from visits based on direct calls received from palliative patients/their families and different palliative care needs, MMSPCT team member provided also two visits according to study protocol, at 1 month and 3 months follow-up period.

#### Control (unexposed) cohort

Palliative patients receiving the usual care in three Croatian counties that have not yet implemented MMSPCT (Karlovac County, Koprivnica-Križevci County and Zagreb County, catering to about 200, 200 and 400–600 palliative care patients each year, respectively). Zagreb County started with MMSPCT teams in July 2018 and after this period contributed to the exposed group too.

Usual care at the primary care level is provided by family physician teams (consist of a primary care physician of family medicine - a general practitioner (GP) and a nurse) in the county health centres (called in Croatia: Dom zdravlja), in collaboration with district nurse. GPs are also required to provide home visits if necessary. During this study, apart from visits based on direct calls received from palliative patients/their families, family physician team member provided also two visits according to study protocol, at month and 3 months follow-up period.

### Outcomes

Several PROMS, PREMS and costs outcomes, related to a Core set (the same outcomes for all integrated care programmes in patients with multimorbidity within the SELFIE project) and the Programme-type specific outcomes for the Palliative Care/oncology programmes were measured: related to I Health/well-being (Activities of daily living, Psychological well-being, Life satisfaction, Social relationship and participation, Resilience, 3-months overall mortality rate, Pain and other symptoms; II Experiences with care (Person-centeredness, Continuity of care, Compassionate care, Preferred place of death) and III Health-care costs (related to the most expensive categories of cost: drugs, medical devices, hospitalisation for acute and chronic care). Definitions, instrument to measure and scale range are described in detail in Table 1.

**Table 1:** Overview of outcomes (Core set and Programme-type specific) (related to Health/well-being, Experiences with care and Resource utilisation and Costs), with definitions, instruments to measure and scale range

Outcome	Definitions/Instrument to measure outcome
I Health/well-being	
Physical functioning	Being in acceptable physical health and being able to do daily activities without needing assistance / Instrument A2. Activities of Daily Living (ADL) (KATZ-15); *Scale: 0–15 (worst)* [22]
Psychological well-being	Absence of stress, worrying, listlessness, anxiety and “feeling down” / Mental Health Inventory (MHI-5); *Scale: 0-100 (best)* [23]
Life satisfaction	Instead of Enjoyment of life (having pleasure in life and being happy**)**, as more appropriate for palliative care patients / Instrument C2. Life satisfaction [24]
Social relationships & participation	Maintaining meaningful connections with others / Impact on Participation & Autonomy (IPA), social life and relationships domain; *Scale: 0–28 (worst)* [25]
Resilience	Successfully recovering from or adjusting to difficulties and restoring one’s equilibrium / Brief Resilience Scale (BRF); *Scale: 6–30 (best)* [26]
Mortality at 3 ^#^	Patient’s medical records
Pain and other symptoms^#^	Physical symptoms like pain, fatigue, nausea/vomiting, dyspnoea, appetite loss, constipation/diarrhoea, insomnia, and quality of life / Special Questionnaire R. Pain and other symptoms were used, Instrument: Quality of Life Questionnaire-Core 15-Palliative care (QLQ-C15-PAL) [27]
II Experiences with care	
Person-centeredness	Ensuring that care matches the individual’s needs, capabilities and preferences and jointly making informed decisions / Person-centered Coordinated Care Experience Questionnaire (P3CEQ), experience of person-centered care domain; *Scale: 0–18 (best)* [7]
Continuity of care	Good collaboration, smooth transitions between caregivers, and no waste of time / Nijmegen Continuity Questionnaire (NCQ), team and cross boundary continuity domain + Client Perceptions of Coordination Questionnaire (CPCQ), item on waiting for appointment /treatment; *Scale: 1–5 (best)* [28, 29]
Compassionate care^#^	Care provided in a warm, sensitive and dignified way with sympathy and respect / The Schwartz Center Compassionate Care Scale; *Scale: 1–10 (best)* [8]
Preferred place of death^#^	Proportion of patients dying at home or the preferred place (and preferred vs. actual place of death)
III Costs	
Health care costs (related to drugs, medical devices, hospitalisation for acute and chronic care)	Croatian Health Insurance Fund data

^#^ Included in the programme-type specific set of outcomes

These outcomes were selected based on a literature review, workshops with representatives from the five stakeholder groups, and focus groups with individuals with multimorbidity, and measured with validated questionnaires [4].

Six items from the Person-Centred Coordinated Care Experiences Questionnaire (P3CEQ) were used to assess the Person-centeredness outcome [7]. The P3CEQ instrument assesses the key domains of the P3C through an 11-item 4-points scale and 5 point-scale questionnaire. Some items assess specifically person-centredness (PC subscale), while some items measure care coordination (CC subscale).

To assess Compassionate care outcome, 12-item scale, the Schwartz Center Compassionate Care Scale was used to measure the compassion provided by care professionals [8]. Patients use the scale to score this care from 1 to 10 (from “not at all successful” to “very successful”).

### Health-care cost

The cumulative health care cost (in Croatian Kuna, HRK), from the payer perspective, related to the most expensive categories of cost: drugs, medical devices, hospitalisation for acute and chronic care, were obtained in June 2019 from the main national insurer in Croatia - Croatian Health Insurance Fund, for included palliative care patients during 3 months follow-up period (Purchasing power parities - PPPs in terms of national currency per US dollar: 3.320 for Croatia in 2019 [30]; exchange rate in terms of national currency per US dollar: 6.623 for Croatia in 2019 [31].

Analysis was done on available palliative care patients’ data, up to 3 months, or up to the date of death, for those who died earlier.

### Data collection - questionnaire

A standardised questionnaire created by compiling multiple validated questionnaires (Appendix 1 in Supplementary Material) was used on outcomes related to health/well-being, experiences with care, resource utilisation and costs. It was administered to patients at the beginning of the study period (T0), after 1 (T1) and after a 3-month period (T2) in both, exposed and control cohorts, by a member of the MMSPCT team or family physician team. The Questionnaire at the T0 visit could have been completed at home (administered by a member of the MMSPCT team or family physician team) or in the hospital (administered by a member of the county hospital palliative care team before discharge and referral to the MMSPCT team or family physician team). After detailed information given about this study, patients who agreed to participate, signed written informed consent. Remuneration to participating patients was not provided. All respective members of the MMSPCT team and family physician team received instructions about the study and all needed documents for conducting the study (e.g., study leaflet, study protocol, informed consent forms, questionnaires) by the Agency for Quality and Accreditation in Health Care and Social Welfare (AAZ) research team.

### Statistical analysis

Propensity score weighting using the subclassification method was used to reduce selection bias by grouping exposed and control group patients into four subclasses based on similar propensity scores. Average treatment effect on the treated (ATT) was estimated using linear regression models on the weighted samples to further adjust for small remaining differences between the groups and increase the efficiency of the estimates. All outcome models were adjusted for age, gender, and baseline value of the same outcome measure, and subclassification weights generated by the MatchIt package were applied as regression weights in all outcome models.

#### Dataset for analysis and method for dealing with missing data

The multiple imputation method was used to obtain valid estimates for missing data (> 20% of data missing in T1), using principal component analysis with the non-linear iterative partial least squares (NIPALS) algorithm. This method was chosen for its lowest imputation error (measured by root-mean-square error, mean absolute error, and Kolmogorov–Smirnov statistic) among 16 different imputation techniques. The methods were tested on our dataset using missCompare R package [32]. We imputed missing data at baseline and at T1 for patients who were alive after T1 (*n* = 317), we then added the data of T2 for those who were alive at T2 and we used this dataset for our analysis.

#### Propensity score estimation and propensity score subclassification method

To control for confounding, we estimated the propensity score, i.e., the conditional probability of receiving treatment [33], for each patient using a logistic model, regressing demographic and baseline covariates on the binary treatment variable (1: palliative treatment, 0: no treatment). Following propensity score estimation, we compared multiple propensity score weighting methods using MatchIt R package [34]. We chose the subclassification method due to its best performance in balancing the covariates, i.e., keeping the absolute standardised mean difference of covariates between − 0.1 and 0.1 after PS weighting.

#### Average treatment effect on the treated (ATT)

Average treatment effect on the treated (ATT) was estimated using linear models with propensity score weighting and clustering based on the subclasses of the propensity score (n of subclasses = 4). Covariates were centred and z-score standardised before running the models to adjust for the differential scaling of variables.

#### Cost outcome variables

Cost outcome variables were analysed using hurdle models due to zero-inflation in the data, first fitting the probability of having a non-zero count and then fitting the value of the count given that it was non-zero. Predictions from the two parts of the hurdle model were combined to estimate the mean costs using the pscl R package. Analyses were performed using different R statistical packages [32, 34]. A p-value of < 0.05 was considered significant.

#### Sample size based on the literature data

The sample size of our analysis was based on the published palliative care studies investigating place of death (150 patients in each group to detect a 14% absolute change in place of death, with 90% power, significance level 0.05; 180 patients in each group to detect a 15% difference, from 50% to 65%, with 80% power, α = 0.05), quality of life (200 patients in each group to detect differences of 0.5 SDs, as moderate change, for which the EORTC QlQ-C30 scores were roughly comparable to a change of 10 units), and/or nausea and pain (110 patients to detect a reduction of 33% in pain and nausea, α = 0.05, allowing for 20% attrition; 50 patients in each group to detect a 10% change in average pain score on a 0–10 point scale, with 90% power, α = 0.05) [35–39]. Based on the literature, the sample size for each group in our study could range from 150 to 200 patients.

#### Reporting

The STROBE guideline was used for reporting this research [40, 41].

## Results

### Patients

**Fig. 1:**
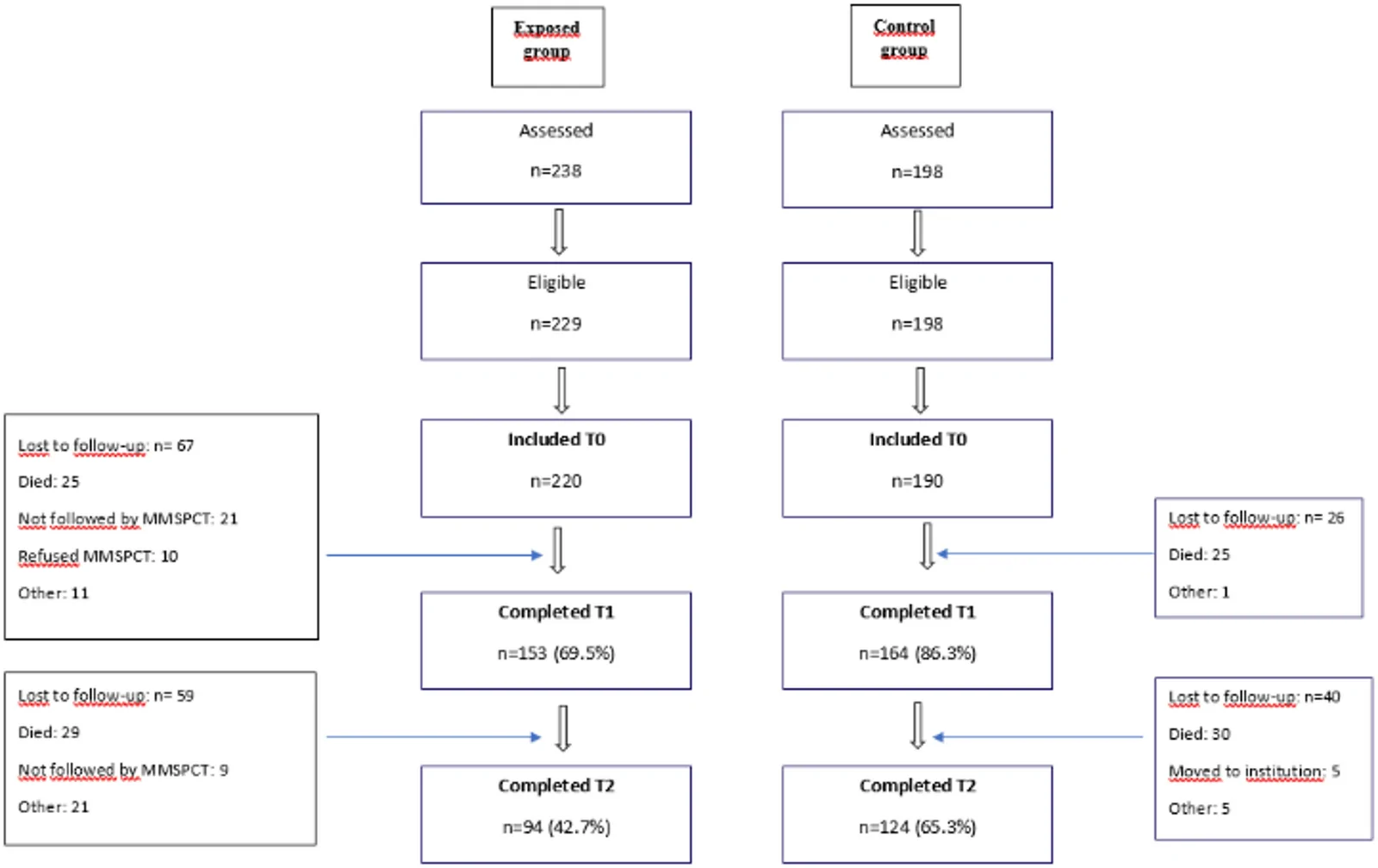
Flow diagram of palliative patients in exposed (MMSPCT) and control (usual care) group

After assessing 436 patients for eligibility by the MMSPCT or primary care physician team, 220 palliative patients were included in the exposed group and 190 in the control group. The flow diagram of recruitment and the main reasons for the loss to follow-up are described in Fig. 1.

153 patients (69.5%) completed T1 (1-month follow-up) in the exposed group and 164 patients (86.3%) in the control group. At T2 (3-month follow-up), 94 patients (42.7%) in the exposed group and 124 patients (65.3%) in the control group completed the study.

Out of 126 patients lost to follow-up during 3-months period in the exposed group, death was the main reason, in 54 (42.9%) patients; protocol deviation in 30 (23.8%) (patients were not followed by the MMSPCT at 1-month and 3-months follow-up period due to misaligned communication between hospital palliative care team and MMSPCT team in one county, or due to turn-over of MMSPCT team members in another county); 10 (7.9%) patients refused the MMSPCT, and in 32 (25.4%) palliative patients other reasons were cause of the loss to follow-up (for example, patients were permanently transferred to the secondary health or social care institutions; moved to other county; or become unresponsive).

In the control group, out of 66 patients lost to follow-up during a 3-month period, most of them died, 25 (37.8%); 5 (7.6%) were moved to other institutions, and in 6 (9.1%), other reasons were the cause of the loss to follow-up.

Table 2 presents the baseline characteristics of palliative patients who were alive at T1 (*n* = 317). We used the 0.1 threshold for absolute standardised mean difference (ASMD) to determine covariate balance [42]. Based on this threshold, the majority of covariates were imbalanced before weighting, except for physical functioning, life satisfaction, fatigue, insomnia, appetite loss, and constipation. After propensity score weighting, the ASMD of all covariates was reduced below the 0.1 threshold, except for living situation (0.271), education level (0.171), marital status (0.228), smoking (0.138), and constipation (0.108). The balance for variables between the two groups is presented in Figure A1, Appendix 2 in Supplementary Material. Although some imbalance remained after propensity score weighting, all outcome models were additionally adjusted for age, sex, and the baseline value of the corresponding outcome, which may have partially reduced residual confounding.

**Table 2:** Baseline characteristics and covariate balance before and after propensity score weighting

Baseline characteristics	Before weighting			After weighting		
	MMSPCT care (*n* = 153)	Usual care (*n* = 164)	SMD	MMSPCT care (*n* = 153)	Usual care (*n* = 164)	SMD
Socio-demographic characteristics						
Age (mean (SD))	72.35 (11.93)	73.88 (11.27)	0.132	72.41 (11.86)	71.83 (12.29)	0.048
Gender = male, n (%)	81 (52.9)	104 (63.4)	0.214	81.0 (52.9)	83.8 (51.1)	0.037
Living situation, n (%)			0.42			0.271
Independent	19 (12.4)	25 (15.2)		19.0 (12.4)	24.7 (15.1)	
With others	133 (86.9)	124 (75.6)		133.0 (86.9)	131.5 (80.2)	
Care or nursing home	1 (0.7)	15 (9.1)		1.0 (0.7)	7.8 (4.7)	
Education level, n (%)			0.256			0.171
Low	127 (83.0)	150 (91.5)		127.0 (83.0)	137.3 (83.7)	
Medium	20 (13.1)	11 (6.7)		20.0 (13.1)	24.6 (15.0)	
High	6 (3.9)	3 (1.8)		6.0 (3.9)	2.1 (1.3)	
Marital status, n (%)			0.44			0.228
Single (never married)	8 (5.2)	4 (2.4)		8.0 (5.2)	4.8 (3.0)	
Married/long-term relationship	92 (60.1)	75 (45.7)		92.0 (60.1)	96.8 (59.0)	
Widow/widower	40 (26.1)	76 (46.3)		40.0 (26.1)	54.6 (33.3)	
Divorced	13 (8.5)	9 (5.5)		13.0 (8.5)	7.8 (4.7)	
Smoking, n (%)			0.251			0.138
Current smoker	18 (11.8)	13 (7.9)		18.0 (11.8)	15.2 (9.3)	
Former smoker	66 (43.1)	57 (34.8)		66.0 (43.1)	64.0 (39.0)	
Never smoked	69 (45.1)	94 (57.3)		69.0 (45.1)	84.8 (51.7)	
Number of health conditions (mean (SD))	6.62 (3.23)	7.02 (3.19)	0.267	6.62 (3.21)	6.50 (2.87)	0.038
Questionnaire outcomes						
Physical functioning, mean (SD)	9.33 (4.74)	9.75 (4.22)	0.093	9.33 (4.74)	9.10 (4.37)	0.05
Psychol. Wellbeing, mean (SD)	55.87 (19.09)	53.02 (17.72)	0.154	55.87 (19.09)	54.62 (17.54)	0.068
Social relations & participation, mean (SD)	9.71 (5.03)	11.64 (4.87)	0.391	9.71 (5.03)	9.94 (5.48)	0.044
Life satisfaction, mean (SD)	2.90 (1.07)	2.80 (0.95)	0.089	2.90 (1.07)	2.94 (1.03)	0.046
Resilience, mean (SD)	19.52 (5.00)	18.05 (5.89)	0.268	19.52 (5.00)	19.45 (5.64)	0.013
Person-centeredness, mean (SD)	13.51 (3.43)	10.76 (4.31)	0.705	13.51 (3.43)	13.36 (4.21)	0.039
Continuity of care, mean (SD)	3.77 (0.79)	3.50 (0.83)	0.342	3.77 (0.79)	3.73 (0.73)	0.051
Compassionate care, mean (SD)	96.22 (23.15)	89.23 (25.21)	0.289	96.22 (23.15)	94.12 (22.70)	0.091
Physical functioning, mean (SD)	22.10 (24.39)	31.44 (30.28)	0.34	27.82 (27.44)	30.31 (29.73)	0.087
Emotional functioning, mean (SD)	60.63 (24.06)	65.98 (27.34)	0.208	67.15 (27.62)	65.83 (24.05)	0.051
Fatigue, mean (SD)	51.75 (24.95)	52.47 (27.73)	0.027	52.50 (26.68)	51.71 (26.07)	0.03
Pain, mean (SD)	49.39 (29.37)	40.66 (30.74)	0.29	41.39 (31.25)	42.74 (28.49)	0.045
Nausea and vomiting, mean (SD)	13.60 (20.52)	16.67 (26.38)	0.13	16.51 (26.12)	16.54 (25.97)	0.001
Dyspnoea, mean (SD)	31.40 (33.98)	26.21 (29.45)	0.163	25.27 (28.10)	24.45 (32.28)	0.027
Insomnia, mean (SD)	38.60 (30.41)	36.97 (30.83)	0.053	34.64 (30.32)	33.81 (29.95)	0.027
Appetite loss, mean (SD)	33.33 (28.77)	35.92 (33.00)	0.084	33.05 (30.48)	32.51 (27.71)	0.018
Constipation, mean (SD)	32.28 (30.27)	32.42 (33.70)	0.004	31.97 (32.65)	28.57 (30.21)	0.108

*MMSPCT *Mobile multidisciplinary specialist palliative care team,* SMD *Absolute Standardised Mean Difference, also called Absolute Standardised Bias

Participants’ health conditions in both cohorts, related to their multimorbidity, are listed in Table A1, Appendix 3 in Supplementary Material.

### Treatment effect

Average treatment effect on the treated (ATT) was estimated on all outcome variables during the T1 visit (after 1 month) and T2 visit (after 3 months) via weighted regression models.

After 1 month of MMSPCT exposure, patients scored significantly better results on two outcomes compared to the control group: one core - person centeredness and one programme-specific outcome - compassionate care (*p* < 0.001) (Table 3). The regression output shows that MMSPCT patients have, on average, a 0.69 (3.83%) unit higher score on person-centeredness and a 3.33 (2.78%) unit higher score on compassionate care, where higher scores indicate better outcomes.

**Table 3:** Regression results for outcomes in T1 (after 1 month) (one core outcome – person centredness and one programme specific outcomes – compassionate care)

	Outcomes	
	Person centeredness	Compassionate care
MMSPCT	0.688***	3.327***
	(0.193)	(1.237)
Person centeredness baseline	0.866***	
	(0.026)	
Compassionate care baseline		0.844***
		(0.027)
Constant	2.130***	15.611***
	(0.744)	(4.751)
Observations	317	317
R2	0.796	0.762
Adjusted R2	0.793	0.759
F Statistic (df = 4; 312)	304.049***	249.238***

*MMSPCT* Mobile multidisciplinary specialist palliative care team, The table shows the coefficients, the standard errors in brackets, and significance levels (*p < 0.1; **p < 0.05; ***p < 0.01); corrected for age and gender

After 3 months of MMSPCT exposure, patients scored significantly better on one core outcome – person-centeredness (*p* < 0.001) and three programme-specific outcomes: physical functioning (*p* < 0.05) and emotional functioning (*p* < 0.05) (measured by Quality of Life Questionnaire-Core 15-Palliative care) and compassionate care (*p* < 0.001) compared to the usual care group (Table 4). They scored 1.87 (10.39%) on person-centeredness, 4.1 (4.1%) points more on physical functioning, 5.18 (5.18%) on emotional functioning, and 6.14 (5.12%) on compassionate care indices on average, compared to the usual care group.

**Table 4:** Regression results for outcomes in T2 (after 3 months) (one Core outcome – Person centredness and three Programme specific outcomes – Compassionate care, Physical functioning and Emotional functioning: both within Pain and other symptoms)

		Outcomes		
	Person centeredness	Compassionate care	Physical functioning	Emotional functioning
MMSPCT	1.874***	6.143***	4.108**	5.181**
	(0.333)	(1.742)	(2.047)	(2.564)
Person centeredness baseline	0.669***			
	(0.044)			
Compassionate care baseline		0.761***		
		(0.037)		
Physical functioning baseline			0.768***	
			(0.034)	
Emotional functioning baseline				0.573***
				(0.050)
Constant	5.925***	25.200***	20.664***	30.958***
	(1.273)	(6.395)	(6.375)	(8.447)
Observations	217	218	215	215
R2	0.603	0.687	0.726	0.417
Adjusted R2	0.595	0.681	0.721	0.406
F Statistic	80.424*** (df = 4; 212)	116.747*** (df = 4; 213)	139.142*** (df = 4; 210)	37.623*** (df = 4; 210)

*MMSPCT* Mobile multidisciplinary specialist palliative care team; The table shows the coefficients, the standard errors in brackets, and significance levels (*p < 0.1; **p < 0.05; ***p < 0.01); corrected for age and gender

In the remaining regression model results related to other core and programme-specific outcomes, the difference between the groups was not significant (Tables A2 to A3, Appendix 4 in Supplementary Material).

Related to the preferred place of death, among patients who died in the exposed group, 94% of patients reported that they would prefer dying at home, and 6% at another place. Out of patients who died, 48% died at their preferred place of death. In the control group, 89.4% of patients preferred dying at home and 10.6% at another place; out of the patients that died, only 23.1% of patients died at their preferred place of death.

Mortality was similar in both groups: during the 3-month follow-up period, 51 (23.2%) patients died in the exposed group compared to 55 (28.9%) patients in the control group.

### Health-care cost

Table 5 presents summaries of healthcare costs by treatment group. The MMSPCT group had lower total, hospitalisation for acute care, and hospitalisation for chronic care costs on average, and slightly higher mean scores for the cost of drugs and medical devices compared to the control group. In the MMSPCT group, patients had 31.5% less healthcare costs on average. After centering the covariates and adjusting for age, sex, baseline costs, and propensity score weights, the average sum of cost was HRK 16,024.41, and HRK 10,463 in the control and MMSPCT groups, respectively. The cost of hospitalisation for acute care is also significantly different between the groups; it is HRK 6,594.53 on average for control patients and HRK 4,654.35 in the MMSPCT group. On the other hand, patients in the MMSPCT group had a higher cost of medical devices than the control group, 31.32% higher on average, amounting to HRK 317.94. The cost of drugs and hospitalisation for chronic care is not significantly different between the two treatment groups. The zero-logit part of the hurdle model shows that there were no significant differences in the likelihood of having a non-zero value for cost, except for the cost of hospitalisation for chronic care, where palliative patients in the MMSPCT group are 78% less likely to have a non-zero cost value (Table 6).

**Table 5:** Summary of health-care cost of the exposed (MMSPCT - Mobile multidisciplinary specialist palliative care team) and control group

Cost category	Group	Min. (HRK)	1st Qu. (HRK)	Median (HRK)	Mean (HRK)	3rd Qu. (HRK)	Max. (HRK)
Drugs	Control group	0.00	733.50	1,357.50	1,961.20	2,502.00	19,005.00
	MMSPCTgroup	0.00	775.00	1,417.00	1,994.00	2,841.00	10,496.00
Medical devices	Control group	0.00	0.00	643.50	1,092.70	1,375.10	12,182.90
	MMSPCT group	0.00	0.00	559.80	1,429.40	1,624.80	14,881.50
Hospitalisation for acute care	Control group	0.00	0.00	0.00	5,870.00	2,625.00	116,475.00
	MMSPCT group	0.00	0.00	0.00	4,555.00	4,275.00	107,400.00
Hospitalisation for chronic care	Control group	0.00	0.00	0.00	2,015.00	0.00	30,141.00
	MMSPCT group	0.00	0.00	0.00	569.10	0.00	28,861.00
Sum of cost	Control group	0.00	1,636.00	3,538.00	10,939.00	10,229.00	123,919.00
	MMSPCT group	0.00	1,725.00	4,096.00	8,597.00	9,945.00	109,854.00

**Table 6:** Regression results for health-care cost outcomes (sum of cost and cost categories: drugs, medical devices, hospitalisation for acute care, hospitalisation for chronic care)

			Health-care Cost outcomes		
	Sum of cost	Cost of Drugs	Cost of Medical devices	Cost of hospitalisation for acute care	Cost of hospitalisation for chronic care
Count model					
MMSPCT	-0.378***	0.102	0.476***	-0.490***	-0.121
	(0.143)	(0.104)	(0.127)	(0.182)	(0.476)
Constant	11.760***	9.392***	7.801***	11.976***	10.232***
	(0.485)	(0.354)	(0.382)	(0.647)	(1.157)
*Zero / logit model*					
MMSPCT	-0.157	-1.448	-0.059	-0.098	-1.527***
	(1.551)	(1.285)	(0.240)	(0.261)	(0.475)
Constant	34.774	11.916**	0.730	1.388	-1.363
	(6,452.442)	(4.838)	(0.794)	(0.853)	(1.332)
Observations	292	293	292	293	293

*MMSPCT* Mobile multidisciplinary specialist palliative care team; *p < 0.1; **p < 0.05; ***p < 0.01; corrected for age and gender

## Discussion

We found that at 1 and 3 months of follow-up, patients exposed to MMSPCT scored significantly higher on person-centeredness and compassionate care outcomes than the usual care group. The same was true for physical functioning and emotional functioning outcomes after 3 months. In the MMSPCT group, more patients died at the preferred place of death. The MMSPCT group has significantly lower measured healthcare costs than the control group.

Our positive results on the two outcomes, person-centeredness and compassionate care, raise the importance of providing such care to palliative patients and measuring these two important outcomes within palliative care research. Although these two outcomes are considered essential for good palliative care at home [43–45], we did not find published data from primary research to compare with our results.

Our better results on physical and emotional functioning after 3 months in the MMSPCT group are in line with the recently published study by Chan et al. [46], which found that emotional symptoms and practical concerns are significantly improved in the first 2 months through a proactive, person-centered, and interdisciplinary approach delivered to 48 home-dwelling patients with advanced diseases in a home care setting. No significant difference between the groups on other measured health/well-being outcomes and other symptoms (i.e., fatigue, pain, nausea, constipation, dyspnoea, insomnia, appetite loss and overall quality of life), is also in line with the latest update of the Cochrane systematic review [6]. Its authors found that the effect of such care on patient outcomes (functional status, psychological well-being, or cognitive status), including control of symptoms, was uncertain [6]. Contrary to our findings, such home-based end-of-life care may slightly improve patient satisfaction at one-month follow-up, but with little or no difference at a six-month follow-up. Some other authors published positive results of home-based end-of-life care on quality of life and care [47, 48], burden of symptoms [49] and the chance of dying at home, if that is the preference [50].

We found that the MMSPCT group had significantly lower healthcare costs in the most expensive categories of cost: drugs, medical devices, hospitalisation for acute and chronic care, which is in line with results from a population-level matched cohort study in Belgium [51] where palliative home care reduces total costs of care at the end of life. Also, early integrated palliative home care can help to avoid hospital admissions and escalation of cost related to the final months of life for people with end-stage diseases [52]. Contrary to our findings, Shepperd et al. found in a Cochrane review that the effect of end-of-life care at home on health service costs was uncertain [6].

In line with other studies, our study showed that more patients exposed to MMSPCT died at their preferred place of death. Shepperd et al. found that people receiving end-of-life care at home were more likely to die at home [6]. In Canada, palliative care near the end-of‐life was associated with an increased likelihood of dying at home [53]. In the UK, 73% of patients known to a specialist community palliative care service, who expressed a choice about their preferred place of death, and 69.3% who wanted to die at home, were able to achieve their preferences [54].

In our study, more than one quarter of patients died within 3 months of the follow-up. This is in line with literature data, which showed that end-stage patients are referred rather late to palliative care [53, 55–58]. According to the study of Quinn et al., published in 2020, palliative care was most commonly initiated late in the course of a person’s disease (≤ 30 days before death, 45.2% of subjects) [53]. Out of all deaths from end-stage chronic obstructive pulmonary disease in 2013, only 37.3% were referred to palliative care, of which 7.2% to palliative home care. For half of the referred patients, the time of onset of palliative care was only six days prior to death [55]. The median timing of referral before death was highest in breast cancer (29 days) and lowest in hematologic cancer patients (10 days) [56]. In the United Kingdom, patients with breast cancer and hematologic cancer were referred 43.5 and 26 days prior to death, respectively [57]. In the United States and in Australia, cancer patients were referred to palliative care 42 days and 54 days prior to death, respectively [58]. In Slovenia, more than half of patients received palliative care provided by the primary health care team for less than one month, and some of them only on the last day [59].

### Limitations and strengths

Our real-world observational study has several limitations, but also strengths. The three-month follow-up period was short due to the late referral to palliative care. The 1-month follow-up attrition rate in our study was higher in the exposed group (> 20%). At the 3-month follow-up, the attrition rate was high in both groups (> 20%), potentially affecting the validity of the estimated difference between groups. As already described, death was the main reason in both groups. Protocol deviation appeared in the exposed group; in 30 (23.8%) patients, the questionnaire was not applied during the 1- and 3-month follow-up period (the majority of these patients were not followed by the MMSPCT at 1-month and 3-month due to misaligned communication between the hospital palliative care team and the MMSPCT team in one county).

Because treatment status was determined at the county level (counties that already implemented MMSPCT vs. those that had not), substantial structural differences may exist between regions. Individual-level propensity score weighting cannot adjust for such county-level confounding. Living situation and educational level of palliative patients remained highly imbalanced after PS weighting, which likely reflects residual county-level differences. Regarding MMSPCT’s experience in providing services, team members from one county have more experience than those in the other two counties. Such differences were not addressed in the sensitivity analyses. On the other hand, a different county setting may lead to better generalisability of our results.

As members of mobile team or primary care physician team applied questionnaire to palliative care patients, this may have influenced participants' responses (e.g., possible patients worries on the impact on further care provided by the teams); an independent trained research assistant who is not a member of the mobile team or primary care physician team would be the best option but the pragmatic approach was used for sustainability of this study.

As palliative care patients under the age of 18 were excluded from this study and may have different needs from adults, extrapolation of the results to children is not possible.

Our cost analysis did not include the operating costs of the MMSPCT itself because these costs were not available at the patient level. Instead, analyses focused on the major healthcare cost categories reimbursed by the Croatian Health Insurance Fund (drugs, medical devices, and hospitalisations). Although the annual reimbursement allocated to standard MMSPCTs and family physician teams in Croatia is broadly comparable, omission of intervention operating costs may have affected the estimated cost differences and should be considered when interpreting these findings.

The strengths of our study are as follows: this was a real-world prospective cohort study that measured a broad range of PROMS and PREMS, as different barriers are recognised in conducting randomized controlled trials in a home-based palliative care [60, 61]. Our questionnaire was based on already validated questionnaires. During the study period, evaluation was performed at different time points, which allows overcoming some challenges recognised with the enrolment of palliative patients [62].

## Conclusion

Based on a cohort study with a 3-month follow-up, MMSPCT at the primary care level may offer added value to palliative patients in Croatia, particularly for important patient outcomes such as person-centeredness, compassionate care, and dying at the preferred place of death. Such care may result in a net saving in healthcare costs on the most expensive categories of cost: drugs, medical devices, hospitalisation for acute and chronic care, compared to the usual care. Further clinical research is needed with a longer follow-up period, as well as further studies of children’s cohorts.

## Supplementary Information

Supplementary Material 1.

## Data Availability

Raw data collected during the study are available from the author Mirjana Huić on request (contact: mirjana.huic@gmail.com).

## Abbreviations

ATT: Average Treatment Effect on the Treated
EU: European Union
GP: General Practitioner
HRK: Croatian Kuna
MMSPCT: Mobile Multidisciplinary Specialist Palliative Care Team
NIPALS: Nonlinear Iterative Partial Least Squares
PPP: Purchasing Power Parities
PREMS: Patient-Reported Experience Measures
PROMS: Patient-Reported Outcome Measures
PSM: Propensity Score Matching
SELFIE: Sustainable intEgrated care models for multi-morbidity, delivery, FInancing, and performance
STROBE: Strengthening the Reporting of Observational Studies in Epidemiology
T0, T1, T2: Time Points
