# Supplementary material for: Added value of a specialized mobile palliative care team compared to the usual care in the primary care setting of Croatia: a 3-month prospective cohort study
Source: BMC Palliat Care. 2026 Jul 25;25:320. doi: 10.1186/s12904-026-02239-1 (PMC13692871; doi:10.1186/s12904-026-02239-1)
Supplement: Supplementary file 1 [file 12904_2026_2239_MOESM1_ESM.docx]

**Appendices 1-5**

**Contents**

[Appendix 1: Inclusion and Exclusion criteria	2](#_Toc187502410)

[Appendix 2: A standardised questionnaire developed for use in the SELFIE project – Palliative care/oncology programmes	3](#_Toc187502411)

[Appendix 3: Statistics on balance for variables between the exposed and the control group	25](#_Toc187502412)

[***Figure A1****. The balance for variables between the exposed and the control group*	25](#_Toc187502413)

[Appendix 4: Summary statistics of health conditions of patients	26](#_Toc187502414)

[***Table A1****. Health conditions in the both study groups with multimorbidity*	26](#_Toc187502415)

[Appendix 5: Regression results for Core and Programme specific outcomes in T1 (after 1 month) and T2 (after 3 months) related to Health/well-being and Experiences with care	27](#_Toc187502416)

[***Table A2.*** *Regression results for outcomes in T1 (after 1 month) - Core outcomes (Physical functioning, Psychological well-being, Life satisfaction, Social relationships & participation, Person-centeredness, Resilience, Continuity of care)*	27](#_Toc187502417)

[***Table A3.*** *Regression results for outcomes in T1 (after 1 month) - Programme specific outcomes (within Pain and other symptoms outcome: Physical functioning, Emotional functioning, Fatigue, Constipation, Pain, Overall Quality of life, Nausea, Dyspnoea, Insomnia, Appetite loss)*	28](#_Toc187502418)

[***Table A4.*** *Regression results for outcomes in T2 (after 3 months) - Core outcomes (Physical functioning, Psychological well-being, Life satisfaction, Social relationships & participation, Person-centeredness, Resilience, Continuity of care)*	29](#_Toc187502419)

[***Table A5****. Regression results for outcomes in T2 (after 3 months) - Programme specific outcomes (within Pain and other symptoms outcome: Physical functioning, Emotional functioning, Fatigue, Constipation, Pain, Overall Quality of life, Nausea, Dyspnoea, Insomnia, Appetite loss)*	30](#_Toc187502420)

# Appendix 1: A standardised questionnaire developed for use in the SELFIE project – Palliative care/oncology programmes

**SELFIE**

**Questionnaire**

Palliative care/oncology programmes

**Instructions**

This questionnaire asks questions about your health and wellbeing, your experience with care and your health care use.

We are interested in your opinion and your experience. There are no right or wrong answers. Choose the answer that best applies to your situation. Mark an X in the box of your choice.

##### Example

1. *Are you male or female?*

**□**	*male*

**☒**	*female (you indicated that you are female)*

If you accidently mark the wrong box, you can make make the box black and mark the correct box.

2. *Are you male or female?*

**☒**	*male ( you have now indicated that you are male)*

■	*female*

For our research, It is important that you answer all questions, even if you find them difficult. Your answers will remain strictly confidential, will not be shared with your careproviders, and will not influence the care you receive.

Completion of the questionnaire will take approximately 25 minutes.

**Many thanks in advance for completing the questionnaire.**

**Part I: Health and well-being**

A. **Daily activities**

*The following items are about activities of daily living. Please mark for each question whether you need help to do these activities. For each question, please indicate if you need help with this activity. Please mark one box for each question.*

|  | **Yes** | **No** |
| --- | --- | --- |
| A1 Do you need help with taking a bath or shower? |  |  |
| A2 Do you need help getting dressed? |  |  |
| A3 Do you need help toileting? |  |  |
| A4 Do you need help sitting down and getting up from a chair? |  |  |
| A5 Do you use incontinence products? |  |  |
| A6 Do you need help with eating? |  |  |
| A7 Do you need help using the telephone? |  |  |
| A8 Do you need help shopping? |  |  |
| A9 Do you need help preparing a meal? |  |  |
| A10 Do you need help taking care of your house? |  |  |
| A11 Do you need help travelling? |  |  |
| A12 Do you need help taking your medications? |  |  |
| A13 Do you need help handling your finances? |  |  |
| A14 Do you need help brushing your hair or shaving? |  |  |
| A15 Do you need help walking about? |  |  |

B. **How you feel**

*These questions are about how you feel and how you have been doing the past month. For each question, please mark the box that best applies to you. Please mark one box for each question.*

| How much of the time during the past month... | All of the time | Most of the time | A good bit of the time | Some of the time | A little of the time | None of the time |
| --- | --- | --- | --- | --- | --- | --- |
| B1 Were you a happy person? | □ | □ | □ | □ | □ | □ |
| B2 Have you felt calm and peaceful? | □ | □ | □ | □ | □ | □ |
| B3 Have you been a very nervous person? | □ | □ | □ | □ | □ | □ |
| B4 Have you felt downhearted and blue? | □ | □ | □ | □ | □ | □ |
| B5 Have you felt so down in the dumps that nothing could cheer you up? | □ | □ | □ | □ | □ | □ |

C. **Life satisfaction**

*Please indicate which statement fits your current situation best by marking ONE box.*

**During the past month, how much of the time have you felt satisfied with your life?**

- Never at all or never
- Rarely
- Sometimes
- Often or most of the time
- Frequently or all of the time

D. **Social relationships**

*The next questions are about the quality and frequency of your social relationships. Please mark one box per question.*

|  | *Very good* | *Good* | *Fair* | *Poor* | *Very Poor* |
| --- | --- | --- | --- | --- | --- |
| D1. My chances of talking to people close to me on equal terms are | □ | □ | □ | □ | □ |
| D2. The quality of my relationships with people who are close to me are | □ | □ | □ | □ | □ |
| D3. The respect I receive from people who are close to me is | □ | □ | □ | □ | □ |
| D4. My relationships with acquaintances are | □ | □ | □ | □ | □ |
| D5. The respect I receive from acquaintances is | □ | □ | □ | □ | □ |
| D6. My chances of having an intimate relationship are | □ | □ | □ | □ | □ |
| D7. My chances of seeing people as often as I want are | □ | □ | □ | □ | □ |

E. **Resilience**

*Below are some statements. Please mark one box per statement.*

|  | *Strongly disagree* | *Disagree* | *Neutral* | *Agree* | *Strongly agree* |
| --- | --- | --- | --- | --- | --- |
| E1. I tend to bounce back quickly after hard times | □ | □ | □ | □ | □ |
| E2. I have a hard time making it through stressful events | □ | □ | □ | □ | □ |
| E3. It does not take me long to recover from a stressful event | □ | □ | □ | □ | □ |
| E4. It is hard for me to snap back when something bad happens | □ | □ | □ | □ | □ |
| E5. I usually come through difficult times with little trouble | □ | □ | □ | □ | □ |
| E6. I tend to take a long time to get over set-backs in my life | □ | □ | □ | □ | □ |

F. **Pain and other symptoms**

*The next questions are about your health situation. Please mark one box per question.*

|  | *Not at all* | *A little* | *Quite a bit* | *Very much* |
| --- | --- | --- | --- | --- |
| F1. Do you have any trouble taking a short walk outside of the house? | □ | □ | □ | □ |
| F2. Do you need to stay in bed or a chair during the day? | □ | □ | □ | □ |
| F3. Do you need help with eating, dressing, washing yourself or using the toilet? | □ | □ | □ | □ |
| *During the past week:* | □ | □ | □ | □ |
| F4. Were you short of breath? | □ | □ | □ | □ |
| F5. Have you had pain? | □ | □ | □ | □ |
| F6. Have you had trouble sleeping? | □ | □ | □ | □ |
| F7. Have you felt weak? | □ | □ | □ | □ |
| F8. Have you lacked appetite? | □ | □ | □ | □ |
| F9. Have you felt nauseated? | □ | □ | □ | □ |
| F10. Have you been constipated? | □ | □ | □ | □ |
| F11. Were you tired? | □ | □ | □ | □ |
| F12. Did pain interfere with your daily activities? | □ | □ | □ | □ |
| F13. Did you felt tense? | □ | □ | □ | □ |
| F14. Did you feel depressed? | □ | □ | □ | □ |

F15. **How would you rate your overall quality of life during the past week?** *Please circle the number between 1 and 7 that best applies to you.*

| **1** | **2** | **3** | **4** | **5** | **6** | **7** |
| --- | --- | --- | --- | --- | --- | --- |
| **Very poor** |  |  |  |  |  | **Excellent** |

G. **Your health today**

*Under each heading, please mark the ONE box that best describes your health TODAY.*

G1. **Mobility**

- I have no problems in walking about
- I have slight problems in walking about
- I have moderate problems in walking about
- I have severe problems in walking about
- I am unable to walk about

G2. **Self-care**

- I have no problems washing and dressing myself
- I have slight problems washing or dressing myself
- I have moderate problems washing or dressing myself
- I have severe problems washing or dressing myself
- I am unable to wash or dress myself

G3. **Usual activities** *(e.g. work, family or leisure activities)*

- I have no problems doing my usual activities
- I have slight problems doing my usual activities
- I have moderate problems doing my usual activities
- I have severe problems doing my usual activities
- I am unable to do my usual activities

G4. **Pain/discomfort**

- I have no pain or discomfort
- I have slight pain or discomfort
- I have moderate pain or discomfort
- I have severe pain or discomfort
- I have extreme pain or discomfort

G5. **Anxiety/depression**

- I am not anxious or depressed
- I am slightly anxious or depressed
- I am moderate anxious or depressed
- I am severely anxious or depressed
- I am extremely anxious or depressed

**Part II: Experience of care**

H. **Person-centeredness**

*The next questions are about your experience and understanding of the care you have received from your health and social care providers in the last month. ‘Care’ could be any treatment or support you received in relation to your health and wellbeing. Please provide a response based on your overall experience if you have received care from more than one service.*

|  | *Not at all* | *To some extent* | *More often than not* | *Always* |
| --- | --- | --- | --- | --- |
| H1. Did you discuss what was most important for YOU in managing your own health and wellbeing? | □ | □ | □ | □ |
| H2. Were you involved as much as you wanted to be in decisions about your care? | □ | □ | □ | □ |
| H3. Were you considered as a ‘whole person’ rather than just a disease/condition in relation to your care? | □ | □ | □ | □ |
| H4. Did your care team / providers involve your family/friends/carers as much as you wanted them to be in decisions about your care? | □ | □ | □ | □ |

H5. **Have you had enough support from your care team / providers to help YOU to manage your own health and wellbeing?**

- I have had no support
- I have not had enough support
- I have had enough support
- I have had a lot of support

H6. **To what extent did you receive useful information at the time you needed it to help you manage your health and wellbeing?**

- I do not receive any information
- I rarely receive enough information
- I sometimes receive enough information
- I always receive enough information
- I receive too much information

I. **Continuity of care**

*The following statements are about the collaboration between caregivers (for example between your general practitioner and your or between your general practitioner and the medical specialist in the hospital). For each statement, please mark the box that best describes how much you agree or whether the statement is not applicable (N/A). Please mark one box per statement.*

|  | *Strongly agree* | *Agree* | *Neutral* | *Disagree* | *Strongly disagree* | *N/A* |
| --- | --- | --- | --- | --- | --- | --- |
| I1. My care providers transfer information very well to one-another | □ | □ | □ | □ | □ | □ |
| I2. My care providers work together very well | □ | □ | □ | □ | □ | □ |
| I3. My care providers are very well connected | □ | □ | □ | □ | □ | □ |
| I4. My care providers always know what one-another is doing | □ | □ | □ | □ | □ | □ |
| I5. I have to wait too long for an appointment or treatment | □ | □ | □ | □ | □ | □ |

J. **Compassionate care**

*Compassionate care involves relating well to patients and families and recognizing and addressing their concerns or distress. Please answer the following questions with regard to the care professional who is in charge of your care. Please use this scale to score this care from 1 to 10.*

| How successfully did your care professional…. | | Not at all successful | | | Somewhat successful | | | | Very successful | | |
| --- | --- | --- | --- | --- | --- | --- | --- | --- | --- | --- | --- |
| J1. | Express sensitivity, caring and compassion for your situation? | 1 | 2 | 3 | 4 | 5 | 6 | 7 | 8 | 9 | 10 |
| J2. | Strive to understand your emotional needs? | 1 | 2 | 3 | 4 | 5 | 6 | 7 | 8 | 9 | 10 |
| J3. | Consider the effect of your illness on you, your family, and the people most important to you? | 1 | 2 | 3 | 4 | 5 | 6 | 7 | 8 | 9 | 10 |
| J4. | Listen attentively to you? | 1 | 2 | 3 | 4 | 5 | 6 | 7 | 8 | 9 | 10 |
| J5. | Convey information to you in a way that was understandable? | 1 | 2 | 3 | 4 | 5 | 6 | 7 | 8 | 9 | 10 |
| J6. | Gain your trust? | 1 | 2 | 3 | 4 | 5 | 6 | 7 | 8 | 9 | 10 |
| J7. | Always involve you in decisions about your treatment? | 1 | 2 | 3 | 4 | 5 | 6 | 7 | 8 | 9 | 10 |
| J8. | Comfortably discuss sensitive, emotional or psychological issues? | 1 | 2 | 3 | 4 | 5 | 6 | 7 | 8 | 9 | 10 |
| J9. | Treat you as a person not just a disease? | 1 | 2 | 3 | 4 | 5 | 6 | 7 | 8 | 9 | 10 |
| J10. | Show respect for you, your family and those important to you? | 1 | 2 | 3 | 4 | 5 | 6 | 7 | 8 | 9 | 10 |
| J11. | Communicate test results in a timely and sensitive manner? | 1 | 2 | 3 | 4 | 5 | 6 | 7 | 8 | 9 | 10 |
| J12. | Spend enough time with you? | 1 | 2 | 3 | 4 | 5 | 6 | 7 | 8 | 9 | 10 |

**Part III: Costs**

K. **Health and social care costs**

*The net questions are about your care utilisation in the last three months. Please add up all control visits, appointments, home visits, and consultations by telephone in the last three months. Telephone calls for making an appointment should not be counted. Please fill in how many consultations you have had approximately if you are unsure about the exact number of consultations.*

|  | ***During the last 3 months, did you visit*** | |
| --- | --- | --- |
| K1. | **……..your general practitioner?** | - **No** - **Yes, namely visit(s)** |
| K2. | **……..a primary care nurse (e.g., practice nurse, nurse practitioner)** | - **No** - **Yes, namely visit(s)** |
| K3. | **……..a GP assistant?** | - **No** - **Yes, namely visit(s)** |
| K4. | **……..a physiotherapist?** | - **No** - **Yes, namely visit(s)** |
| K5. | **……..an occupational therapist?** | - **No** - **Yes, namely visit(s)** |
| K6. | **……..a** **speech therapist?** | - **No** - **Yes, namely visit(s)** |
| K7. | **……..a dietician?** | - **No** - **Yes, namely visit(s)** |
| K8. | **……..a podiatrist?** | - **No** - **Yes, namely visit(s)** |
| K9. | **……..a medical specialist (e.g., geriatrician, lung specialist, cardiologist, eye specialist, rheumatologist, neurologist, elderly care physician)** Add up all appointments. | - **No** - **Yes, namely visit(s)** |
| K10. | **……..a psychologist, psychiatrist or psychotherapist?** Add up all appointments. | - **No** - **Yes, namely visit(s)** |
| K11. | **……..a district nurse or community nurse?** Add up all appointments. | - **No** - **Yes, namely visit(s)** |
| K12. | **……..a social worker?** | - **No** - **Yes, namely visit(s)** |
| K13. | **……..a welfare worker?** | - **No** - **Yes, namely visit(s)** |
| K14. | **……..a homoeopathist or an acupuncturist?** Add up all appointments. | - **No** - **Yes, namely visit(s)** |
| K15. | **……..dentist?** | - **No** - **Yes, namely visit(s)** |
| K16. | **……..optician?** | - **No** - **Yes, namely visit(s)** |
| K17. | **……..other, namely:** | - **No** - **Yes, namely visit(s)** |
| K18. | **……..other, namely:** | - **No** - **Yes, namely visit(s)** |
| K19. | **……..other, namely:** | - **No** - **Yes, namely visit(s)** |

|  | ***During the last 3 months did you visit any of the following services below, and if so, how often? Please only fill in day-visits (outpatient), and not overnight stays (inpatient)*** | |
| --- | --- | --- |
| K20. | **……..residential care or nursing home** | - **No** - **Yes, namely visit(s)** |
| K21. | **……..rehabilitation center** | - **No** - **Yes, namely visit(s)** |
| K22. | **……..other, namely:** | - **No** - **Yes, namely visit(s)** |

K23. **During the last 3 months, did you visit a hospital emergency room?**

- No
- Yes, namely	time(s)

K24. **Have you been admitted to a hospital in the past 3 months?**

- No
- Yes, namely	days in total *(add up all days in case of more admissions).*

K25. **Were you admitted elsewhere because of your health during the last 3 months?** *For example you stayed in a home for residential care or a nursing home, in a psychiatric hospital or rehabilitation center.*

- No *→ Go to question K29*
- Yes

|  | ***During the last 3 months, I have been admitted to a...*** | |
| --- | --- | --- |
| K26. | **……..residential care or nursing home** | - **No** - **Yes, namely days in the past**   **three months** |
| K27. | **……..rehabilitation center** | - **No** - **Yes, namely days in the past**   **three months** |
| K28. | **……..psychiatric hospital** | - **No** - **Yes, namely days in the past**   **three months** |

K29. **Did you receive home care in the last 3 months?**

- No *→ Go to question* *K33*
- Yes

|  | ***During the last 3 months (13 weeks), did you receive the following home care services…*** | |
| --- | --- | --- |
| K30. | **……..housekeeping and home help** *(e.g., vacuum cleaning, doing the laundry, making up your bed, going for daily groceries)* | - **No** - **Yes, namely hours a week**   **weeks during the last 3 months** |
| K31. | **……..personal care** *(e.g., help for bathing or help to get dressed)* | - **No** - **Yes, namely hours a week**   **weeks during the last 3 months** |
| K32. | **……..nursing** *(e.g., put on a bandage, administer medicines, measure blood pressure)* | - **No** - **Yes, namely hours a week**   **weeks during the last 3 months** |

K33. **Did you take any medication during the last 3 months?**

- Yes
- No **→** *Go to question L1*

K34. **What medication did you take during the last 3 months?**

*With medication we mean all drugs that were prescribed and medication that you have bought at the pharmacy or a drugstore. We have given 2 examples.*

***Note:*** *Filling in the questions below will be made much easier if you look at your medication boxes. You’ll find the dose you have to take per time. And how often you have to do so per day.* ***Did you take more or less?*** *Please fill in the dose that you have actually taken.*

| ***What is the medication name?*** | ***How much did you take per time?***  *See the box or the package.* | ***How many times did you take this per day?***  *See the box or the package.* | ***How many days during the past 3 months did you take the medication?*** |
| --- | --- | --- | --- |
| *example 1*  Metoprolol (blood pressure) | *example*  100mg | *example*  1 time | *example*  90 days |
| *example 2*  Furosemide (diuretic) | *example*  40 mg | *example*  1 time | *example*  26 days  (2 times a week; 13 weeks) |
|  |  |  |  |
|  |  |  |  |
|  |  |  |  |
|  |  |  |  |
|  |  |  |  |

L. **Informal caregiving**

|  | *Please indicate if in the past three months (13 weeks) you received the following help from family or other relatives due to your health condition. If yes, how often?* | |
| --- | --- | --- |
| L1. | **…….. household activities?**  *For example, food preparation, cleaning, gardening, and taking care of and playing with your (grand)children* | - **No** - **Yes, namely hours a week**   **weeks in the**  **past 3 months** |
| L2. | **……..personal care?**  *For example, dressing, washing, shaving, going to the toilet, eating, drinking and taking medication* | - **No** - **Yes, namely hours a week**   **weeks in the**  **past 3 months** |
| L3. | **……..practical support?**  *For example, mobility outside the house including assistance with walking or wheelchair, visiting family or friends, seeing health care contacts (e.g., doctors’ appointment), and taking care of financial matters* | - **No** - **Yes, namely hours a week**   **weeks in the**  **past 3 months** |

**Part IV: Demographics**

M1. **What is the date today?**

| D | D |  | M | M |  | Y | Y | Y | Y |
| --- | --- | --- | --- | --- | --- | --- | --- | --- | --- |
|  |  |  |  |  |  |  |  |  |  |

M2. **What is your date of birth?**

| D | D |  |  | M | M |  | Y | Y | Y | Y |
| --- | --- | --- | --- | --- | --- | --- | --- | --- | --- | --- |
|  |  |  |  |  |  |  |  |  |  |  |

M3. **What is your gender?**

- Male
- Female

M4. **What is the highest degree or level of school that you have completed?**

- Early childhood education
- Primary education
- Lower secondary education
- Upper secondary education
- Post-secondary non-tertiary education
- Tertiary education
- Short-cycle tertiary education
- Bachelor’s or equivalent level
- Master’s or equivalent level
- Doctoral or equivalent level.

M5. **What is your marital status:**

- Single (never married)
- Married / long-term relationship
- Widow / widower
- Divorced

M6. **What is your living situation:**

- Independent, alone
- With others (partner, children, etc.)
- Care home / residential care centre since ……. /…….. /……. (DD/MM/YY)
- Nursing home since ……. /…….. /……. (DD/MM/YY)

M7. **Which of the following statements about occupational status apply to you?** *(multiple answers allowed)*

- I have a paid job
- I do volunteer work
- I am retired or on pre-pension
- I am work disabled, for …………….. %
- I am looking for a job
- I have a paid job
- I am a housewife / househusband
- I am a student

M8. **What is your smoking status?**

- Current smoker
- Former smoker
- Never smoker

**Health conditions**

*The following is a list of common health problems. Please indicate for each condition if you have this or not. At the bottom there is space to list health problems not already presented.*

| Have you have this condition? | *No* | | *Yes* |
| --- | --- | --- | --- |
| M9. Asthma | □ | | □ |
| M10. Cancer during the past five years (not including small skin cancers) | □ | | □ |
| M11. Chronic back pain or sciatica | □ | | □ |
| M12. Chronic bronchitis, COPD or emphysema | □ | | □ |
| M13. Chronic kidney disease | □ | | □ |
| M14. Colon problem, irritable bowel or colitis | □ | | □ |
| M15. Congestive heart failure | □ | | □ |
| M16. Depression, anxiety or emotional difficulties | □ | | □ |
| M17. Diabetes | □ | | □ |
| M18. Hard of hearing | □ | | □ |
| M19. Heart disease, angina (chest pain from heart problem), heart attack, bypass surgery or angioplasty | □ | | □ |
| M20. High blood pressure | □ | | □ |
| M21. High cholesterol | □ | | □ |
| M22. Osteoarthritis (not rheumatoid arthritis) | □ | | □ |
| M23. Osteoporosis (thinning of the bones) | □ | | □ |
| M24. Overweight | □ | | □ |
| M25. Poor circulation in your legs | □ | | □ |
| M26. Rheumatoid arthritis | □ | | □ |
| M27. Rheumatic disease, fibromyalgia or lupus | □ | | □ |
| M28. Stomach problem, ulcer, gastritis or reflux | □ | | □ |
| M29. Stroke | □ | | □ |
| M30. Thyroid disorder | □ | | □ |
| M31. Vision problem | □ | | □ |
| M32. Other (please write in) |  |  | |

**This is the end of the questionnaire.**

**Thank you for completing the questionnaire!!!**

***************Information extracted from REGISTRATION data ***************

**Mortality**

**Mortality**

*“x-month overall mortality rate (where x is different for palliative care and oncology programmes)”*

**Timely access to care**

**Timely access to care**

*“Number of days between referral and start of treatment or care of interest”*

**Preferred place of death**

**Preferred place of death**

*“Proportion of patients dying at home or the preferred place”*

****This concept could be measured by asking the care providers the following questions****

- **Where did the patient die? ..….**
- **Did they ever say that there was a place they would like to die?**

– If yes, where was the place?

– If no, where do you think that they would have wanted to die?

# Appendix 2: Statistics on balance for variables between the exposed and the control group

## ***Figure A1****. The balance for variables between the exposed and the control group*

# Appendix 3: Summary statistics of health conditions of patients

## ***Table A1****. Health conditions in the both study groups with multimorbidity*

| **Health condition** | **Control** | | | **Exposed** | | |
| --- | --- | --- | --- | --- | --- | --- |
|  | **N** | **%** | **% Missing** | **N** | **%** | **% Missing** |
| Cancer during the past five years (not including small skin cancers) | 98 | 59.76% | 1.83% | 105 | 68.63% | 0.65% |
| Poor circulation in your legs | 106 | 64.63% | 0.61% | 92 | 60.13% | 1.31% |
| Vision problem | 87 | 53.05% | 1.22% | 73 | 47.71% | 1.31% |
| High blood pressure | 98 | 59.76% | 0.61% | 72 | 47.06% | 1.31% |
| Chronic back pain or sciatica | 88 | 53.66% | 1.22% | 64 | 41.83% | 0.65% |
| Stomach problem, ulcer, gastritis or reflux | 80 | 48.78% | 1.22% | 57 | 37.25% | 0.65% |
| Colon problem, irritable bowel or colitis | 35 | 21.34% | 1.83% | 51 | 33.33% | 0.65% |
| High cholesterol | 32 | 19.51% | 0.61% | 47 | 30.72% | 0.65% |
| Depression, anxiety or emotional difficulties | 57 | 34.76% | 1.22% | 46 | 30.07% | 0.65% |
| Diabetes | 39 | 23.78% | 0.61% | 44 | 28.76% | 1.31% |
| Hard of hearing | 53 | 32.32% | 0.61% | 42 | 27.45% | 1.31% |
| Heart disease, angina (chest pain from heart problem), heart attack, bypass surgery or angioplasty | 45 | 27.44% | 0.61% | 39 | 25.49% | 0.65% |
| Thyroid disorder | 21 | 12.80% | 0.61% | 33 | 21.57% | 1.31% |
| Chronic bronchitis, COPD or emphysema | 29 | 17.68% | 0.61% | 30 | 19.61% | 0.65% |
| Osteoporosis (thinning of the bones) | 37 | 22.56% | 1.22% | 30 | 19.61% | 0.65% |
| Chronic kidney disease | 32 | 19.51% | 1.22% | 29 | 18.95% | 1.31% |
| Overweight | 26 | 15.85% | 1.22% | 28 | 18.30% | 0.65% |
| Congestive heart failure | 31 | 18.90% | 1.83% | 21 | 13.73% | 0.65% |
| Stroke | 36 | 21.95% | 1.22% | 20 | 13.07% | 0.65% |
| Osteoarthritis (not rheumatoid arthritis) | 35 | 21.34% | 1.22% | 18 | 11.76% | 0.65% |
| Rheumatoid arthritis | 10 | 6.10% | 1.22% | 15 | 9.80% | 0.65% |
| Asthma | 15 | 9.15% | 1.22% | 10 | 6.54% | 0.65% |
| Rheumatic disease, fibromyalgia or lupus | 6 | 3.66% | 1.22% | 9 | 5.88% | 0.65% |

# Appendix 4: Regression results for Core and Programme specific outcomes in T1 (after 1 month) and T2 (after 3 months) related to Health/well-being and Experiences with care

## ***Table A2.*** *Regression results for outcomes in T1 (after 1 month) - Core outcomes (Physical functioning, Psychological well-being, Life satisfaction, Social relationships & participation, Person-centeredness, Resilience, Continuity of care)*

|  |  |  | **Outcomes** |  |  |  |  |
| --- | --- | --- | --- | --- | --- | --- | --- |
|  | **Physical functioning** (Katz ADL) | **Psychological well-being** | **Life satisfaction** | **Social relationships & participation** | **Person-centeredness** | **Resilience** | **Continuity of care** |
| **MMSPCT** | -0.287 | -1.109 | -0.003 | 0.237 | **0.688***** | -0.226 | 0.027 |
|  | (0.199) | (1.369) | (0.072) | (0.295) | **(0.193)** | (0.351) | (0.042) |
| Physical functioning baseline | 0.960*** |  |  |  |  |  |  |
|  | (0.023) |  |  |  |  |  |  |
| Psychological well-being baseline |  | 0.738*** |  |  |  |  |  |
|  |  | 0.038) |  |  |  |  |  |
| Life satisfaction baseline |  |  | 0.825*** |  |  |  |  |
|  |  |  | (0.036) |  |  |  |  |
| Social relationships & participation baseline |  |  |  | 0.929*** |  |  |  |
|  |  |  |  | (0.030) |  |  |  |
| Person-centeredness baseline |  |  |  |  | 0.866*** |  |  |
|  |  |  |  |  | (0.026) |  |  |
| Resilience baseline |  |  |  |  |  | 0.829*** |  |
|  |  |  |  |  |  | (0.034) |  |
| Continuity of care baseline |  |  |  |  |  |  | 0.858*** |
|  |  |  |  |  |  |  | (0.028) |
| Constant | 0.359 | 18.962*** | 0.166 | 0.752 | 2.130*** | 6.196*** | 0.464** |
|  | (0.611) | (4.909) | (0.274) | (0.904) | (0.744) | (1.329) | (0.183) |
| **Observations** | **317** | **317** | **317** | **317** | **317** | **317** | **317** |
| R2 | 0.862 | 0.558 | 0.644 | 0.780 | 0.796 | 0.675 | 0.752 |
| Adjusted R2 | 0.860 | 0.552 | 0.639 | 0.778 | 0.793 | 0.671 | 0.749 |
| F Statistic (df = 4; 312) | 485.952*** | 98.300*** | 140.914*** | 277.178*** | 304.049*** | 162.205*** | 236.158*** |

Note: The table shows the coefficients, the standard errors in brackets, and significance levels (*p<0.1; **p<0.05; ***p<0.01); corrected for age and gender

## ***Table A3.*** *Regression results for outcomes in T1 (after 1 month) - Programme specific outcomes (within Pain and other symptoms outcome: Physical functioning, Emotional functioning, Fatigue, Constipation, Pain, Overall Quality of life, Nausea, Dyspnoea, Insomnia, Appetite loss)*

|  |  |  |  | **Outcomes** |  |  |  |  |  |  |
| --- | --- | --- | --- | --- | --- | --- | --- | --- | --- | --- |
|  | **Physical functioning** | **Emotional functioning** | **Fatigue** | **Constipation** | **Pain** | **Overall Quality of life** | **Nausea** | **Dyspnoea** | **Insomnia** | **Appetite loss** |
| **MMSPCT** | 0.207 | 0.614 | 1.770 | 1.082 | -3.251 | 2.507 | 0.719 | 1.049 | 0.954 | 1.432 |
|  | (1.193) | (1.851) | (1.903) | (2.108) | (2.295) | (1.847) | (2.304) | (2.058) | (2.289) | (2.375) |
| Physical functioning baseline | 0.896*** |  |  |  |  |  |  |  |  |  |
|  | (0.021) |  |  |  |  |  |  |  |  |  |
| Emotional functioning baseline |  | 0.752*** |  |  |  |  |  |  |  |  |
|  |  | (0.036) |  |  |  |  |  |  |  |  |
| Fatigue baseline |  |  | 0.684*** |  |  |  |  |  |  |  |
|  |  |  | (0.037) |  |  |  |  |  |  |  |
| Constipation baseline |  |  |  | 0.715*** |  |  |  |  |  |  |
|  |  |  |  | (0.034) |  |  |  |  |  |  |
| Pain baseline |  |  |  |  | 0.715*** |  |  |  |  |  |
|  |  |  |  |  | (0.039) |  |  |  |  |  |
| Overall Quality of life baseline |  |  |  |  |  | 0.707*** |  |  |  |  |
|  |  |  |  |  |  | (0.039) |  |  |  |  |
| Nausea baseline |  |  |  |  |  |  | 0.621*** |  |  |  |
|  |  |  |  |  |  |  | (0.045) |  |  |  |
| Dyspnoea baseline |  |  |  |  |  |  |  | 0.735*** |  |  |
|  |  |  |  |  |  |  |  | (0.034) |  |  |
| Insomnia baseline |  |  |  |  |  |  |  |  | 0.705*** |  |
|  |  |  |  |  |  |  |  |  | (0.038) |  |
| Appetite loss baseline |  |  |  |  |  |  |  |  |  | 0.821*** |
|  |  |  |  |  |  |  |  |  |  | (0.041) |
| Constant | 3.153 | 25.356*** | 12.373** | -3.388 | 4.135 | 17.621*** | -5.795 | -8.439 | 5.931 | -0.552 |
|  | (3.917) | (6.244) | (6.106) | (6.503) | (7.073) | (6.042) | (7.194) | (6.394) | (7.151) | (7.337) |
| **Observations** | **317** | **317** | **317** | **317** | **317** | **317** | **317** | **317** | **317** | **317** |
| R2 | 0.856 | 0.589 | 0.539 | 0.598 | 0.535 | 0.521 | 0.391 | 0.603 | 0.527 | 0.567 |
| Adjusted R2 | 0.854 | 0.583 | 0.533 | 0.593 | 0.529 | 0.515 | 0.383 | 0.598 | 0.521 | 0.562 |
| F Statistic (df = 4; 312) | 464.887*** | 111.598*** | 91.044*** | 116.054*** | 89.803*** | 84.813*** | 50.089*** | 118.572*** | 86.925*** | 102.220*** |

Note: The table shows the coefficients, the standard errors in brackets, and significance levels (*p<0.1; **p<0.05; ***p<0.01); corrected for age and gender

## ***Table A4.*** *Regression results for outcomes in T2 (after 3 months) - Core outcomes (Physical functioning, Psychological well-being, Life satisfaction, Social relationships & participation, Person-centeredness, Resilience, Continuity of care)*

|  |  |  | **Outcomes** |  |  |  |  |
| --- | --- | --- | --- | --- | --- | --- | --- |
|  | **Physical functioning** (Katz ADL) | **Psychological well-being** | **Life satisfaction** | **Social relationships & participation** | **Person-centeredness** | **Resilience** | **Continuity of care** |
| **MMSPCT** | -0.500 | 3.647* | 0.064 | -0.772 | **1.874***** | 0.105 | 0.047 |
|  | (0.320) | (2.015) | (0.106) | (0.483) | **(0.333)** | (0.592) | (0.065) |
| Physical functioning baseline | 0.962*** |  |  |  |  |  |  |
|  | (0.036) |  |  |  |  |  |  |
| Psychological well-being baseline |  | 0.639*** |  |  |  |  |  |
|  |  | (0.054) |  |  |  |  |  |
| Life satisfaction baseline |  |  | 0.625*** |  |  |  |  |
|  |  |  | (0.050) |  |  |  |  |
| Social relationships & participation baseline |  |  |  | 0.850*** |  |  |  |
|  |  |  |  | (0.049) |  |  |  |
| Person-centeredness baseline |  |  |  |  | 0.669*** |  |  |
|  |  |  |  |  | (0.044) |  |  |
| Resilience baseline |  |  |  |  |  | 0.671*** |  |
|  |  |  |  |  |  | (0.055) |  |
| Continuity of care baseline |  |  |  |  |  |  | 0.749*** |
|  |  |  |  |  |  |  | (0.045) |
| Constant | -0.136 | 19.774*** | 1.718*** | -0.381 | 5.925*** | 10.158*** | 0.700** |
|  | (0.958) | (6.995) | (0.386) | (1.440) | (1.273) | (2.146) | (0.278) |
| **Observations** | **218** | **217** | **215** | **218** | **217** | **218** | **210** |
| R2 | 0.790 | 0.418 | 0.472 | 0.642 | 0.603 | 0.443 | 0.589 |
| Adjusted R2 | 0.786 | 0.407 | 0.462 | 0.635 | 0.595 | 0.432 | 0.581 |
| F Statistic | 200.466*** (df = 4; 213) | 38.025*** (df = 4; 212) | 46.853*** (df = 4; 210) | 95.531*** (df = 4; 213) | 80.424*** (df = 4; 212) | 42.334*** (df = 4; 213) | 73.324*** (df = 4; 205) |

Note: The table shows the coefficients, the standard errors in brackets, and significance levels (*p<0.1; **p<0.05; ***p<0.01); corrected for age and gender

## ***Table A5****. Regression results for outcomes in T2 (after 3 months) - Programme specific outcomes (within Pain and other symptoms outcome: Physical functioning, Emotional functioning, Fatigue, Constipation, Pain, Overall Quality of life, Nausea, Dyspnoea, Insomnia, Appetite loss)*

|  |  |  |  | **Outcomes** |  |  |  |  |  |  |
| --- | --- | --- | --- | --- | --- | --- | --- | --- | --- | --- |
|  | **Physical functioning** | **Emotional functioning** | **Fatigue** | **Constipation** | **Pain** | **Overall Quality of life** | **Nausea** | **Dyspnoea** | **Insomnia** | **Appetite loss** |
| **MMSPCT** | **4.108**** | **5.181**** | 0.614 | -5.681 | -2.736 | 4.355 | -1.741 | -3.246 | 0.096 | -2.990 |
|  | **(2.047)** | **(2.564)** | (3.108) | (3.593) | (3.444) | (2.766) | (2.956) | (3.118) | (3.506) | (3.763) |
| Physical functioning baseline | 0.768*** |  |  |  |  |  |  |  |  |  |
|  | (0.034) |  |  |  |  |  |  |  |  |  |
| Emotional functioning baseline |  | 0.573*** |  |  |  |  |  |  |  |  |
|  |  | (0.050) |  |  |  |  |  |  |  |  |
| Fatigue baseline |  |  | 0.579*** |  |  |  |  |  |  |  |
|  |  |  | (0.060) |  |  |  |  |  |  |  |
| Constipation baseline |  |  |  | 0.400*** |  |  |  |  |  |  |
|  |  |  |  | (0.058) |  |  |  |  |  |  |
| Pain baseline |  |  |  |  | 0.515*** |  |  |  |  |  |
|  |  |  |  |  | (0.058) |  |  |  |  |  |
| Overall Quality of life baseline |  |  |  |  |  | 0.593*** |  |  |  |  |
|  |  |  |  |  |  | (0.057) |  |  |  |  |
| Nausea baseline |  |  |  |  |  |  | 0.250*** |  |  |  |
|  |  |  |  |  |  |  | (0.058) |  |  |  |
| Dyspnoea baseline |  |  |  |  |  |  |  | 0.562*** |  |  |
|  |  |  |  |  |  |  |  | (0.049) |  |  |
| Insomnia baseline |  |  |  |  |  |  |  |  | 0.506*** |  |
|  |  |  |  |  |  |  |  |  | (0.060) |  |
| Appetite loss baseline |  |  |  |  |  |  |  |  |  | 0.476*** |
|  |  |  |  |  |  |  |  |  |  | (0.067) |
| Constant | 20.664*** | 30.958*** | -4.588 | -2.867 | 7.750 | 44.180*** | -3.429 | 1.467 | 16.019 | 0.573 |
|  | (6.375) | (8.447) | (9.709) | (10.801) | (10.160) | (8.824) | (8.997) | (9.422) | (10.625) | (11.183) |
| **Observations** | **215** | **215** | **214** | **215** | **215** | **215** | **217** | **217** | **216** | **217** |
| R2 | 0.726 | 0.417 | 0.359 | 0.206 | 0.310 | 0.400 | 0.093 | 0.393 | 0.254 | 0.217 |
| Adjusted R2 | 0.721 | 0.406 | 0.346 | 0.190 | 0.297 | 0.389 | 0.076 | 0.382 | 0.240 | 0.203 |
| F Statistic | 139.142*** (df = 4; 210) | 37.623*** (df = 4; 210) | 29.230*** (df = 4; 209) | 13.583*** (df = 4; 210) | 23.590*** (df = 4; 210) | 35.002*** (df = 4; 210) | 5.443*** (df = 4; 212) | 34.367*** (df = 4; 212) | 17.932*** (df = 4; 211) | 14.717*** (df = 4; 212) |

Note: The table shows the coefficients, the standard errors in brackets, and significance levels (*p<0.1; **p<0.05; ***p<0.01); corrected for age and gender
